# Left behind, not alone: feeling, function and neurophysiological markers of self-expansion among left-behind children and not left-behind peers

**DOI:** 10.1093/scan/nsaa062

**Published:** 2020-05-04

**Authors:** Chongzeng Bi, Daphna Oyserman, Ying Lin, Jiyuan Zhang, Binghua Chu, Hongsheng Yang

**Affiliations:** 1 Faculty of Psychology, Southwest University, Chongqing 400715, China; 2 Department of Psychology, SGM 803 3620 South McClintock Ave, University of Southern California, Los Angeles, CA 90089, USA

**Keywords:** left-behind children, interdependence, self-concept, self-reference effect, event-related potentials (ERPs)

## Abstract

Four in 10 young rural Chinese children are ‘left behind’ by parents migrating for economic opportunities. Left-behind children do as well academically and imagine as many possible futures for themselves as their peers, implying that they must compensate in some ways for loss of everyday contact with their parents. Three studies test and find support for the prediction that compensation entails self-expansion to include a caregiving grandmother rather than one’s mother in self-concept, as is typical in Chinese culture. We measured self-expansion with feeling, function and neurophysiological variables. Twelve-year-old middle school left-behind children (Study 1, *N* = 66) and 20-year-old formerly left-behind children (now in college, Studies 2 and 3, *N* = 162) felt closer to their grandmothers and not as close to their mothers as their peers. Self-expansion had functional consequence (spontaneous depth-of-processing) and left a neurophysiological trace (event-related potential, Study 3). Left-behind participants had enhanced recall for information incidentally connected to grandmothers (Studies 1 and 3, not Study 2). Our results provide important insights into how left-behind children cope with the loss of parental presence: they include their grandmother in their sense of self. Future studies are needed to test downstream consequences for emotional and motivational resilience.

**Highlights**

1. Four in 10 rural Chinese children are left behind by parents seeking economic opportunity.
2. These ‘left-behind’ (LB) children cope by including grandmother in their sense of self.
3. This effects spontaneous elaboration of information incidentally linked to grandmothers.
4. Neurological (P3, in ERP) and memory effects converge.

## Introduction

A staggering number of parents worldwide leave their children to seek work—about 1 in 5 rural children worldwide (Duan and Wu, 2009), over a quarter of children in the Philippines and about 4 in 10 in China, Ecuador and South Africa (Fellmeth *et al.*, 2018). In China, this constitutes 61 million left-behind (LB) rural Chinese children (All-Woman China Federation [ACWF], 2013). Chinese parents often leave as soon as their children start school, their inflexible work schedules and high travel cost make visits rare, so they try to sustain relationships via telecommunication (Pan *et al.*, 2013). Being LB impairs children’s emotional (e.g. Wong *et al.*, 2009; Jia and Tian, 2010; Dai and Chu, 2017; Wang *et al.*, 2019b) and physical health (Gao *et al.*, 2010; Huang *et al.*, 2015; Griffiths *et al.*, 2018). But parents hope this sacrifice pays off in better opportunities and outcomes for their children (ACWF, 2013; Griffiths *et al.*, 2018). There is some evidence that Chinese LB children understand the choice made by their parents and accept their situation (Dai and Chu, 2018). As a testament to the importance of parents, when asked who the most important person in their life is, 81% of LB children chose their parents, the rest (19%) chose their grandparents (Ye and Pan, 2011). We build on prior studies suggesting that children left in the care of grandparents (most commonly grandmothers) have robust school-focused future identities and do as well academically as their peers (Bi and Oyserman, 2015). We predict that they do so by modifying a common psychological resource—self-expansion to include a close other, including their grandmothers in their sense of self to compensate for the loss of mothers in this role.

We focus narrowly on mothers and grandmothers for theoretical and empirical reasons: Chinese culture emphasizes the deep connection between children and mothers (Ray *et al.*, 2010) and the importance of grandparents (Wang *et al.*, 2019a). Empirically, maternal absence is particularly difficult for LB children (Xu *et al.*, 2019), and when parents leave, grandmothers are the most likely caregivers (Fan *et al.*, 2010). A narrow focus increases the clarity of results. At the same time, our findings might apply to anyone who takes on the main caretaking role. In China, people who are connected through generational bonds and frequent interaction are the ones likely to be included in the self through self-expansion (Tan *et al.*, 2015).

We address four gaps in the literature: is inclusion of grandmother in self larger for LB than other children, is this inclusion a compensation for loss of mother or in addition to inclusion of mother, is this pattern stable and does it have functional and neurophysiological consequence. First, regarding differential patterns of inclusion of grandmother, we provide evidence that LB children uniquely include grandmothers in their sense of self compared to NLB children. Second, regarding differential patterns of inclusion of mother, we provide evidence that grandmother inclusion is to compensate for inclusion of mother in self. Third, regarding stability over time, we provide evidence that this pattern may remain after children become young adults and leave home. Fourth, regarding functional and neurophysiological consequences, we examine consequences for cognitive processing (operationalized as enhanced incidental recall) and look for a neurological signature of enhanced attention response [operationalized as P3 in event-related potential (ERP)]. Before describing our studies and results, we summarize prior literature on the benefits of self-expansion to include others in the self, how this is measured and its cognitive and neurological signatures.

## Defining and conceptualizing self-expansion

James (1890) coined the term self-expansion to describe people’s tendency to include other people and things in their sense of self. Currently, self-expansion is conceptualized as a feeling of closeness, connection and overlap with another person (Aron *et al.*, 1992, 2013; Aron and Fraley, 1999). It is understood to be a social resource in two ways. First, it provides access to the included other person’s traits, goals, actions and resources (Mashek *et al.*, 2003; Aron *et al.*, 2013). Second, it provides validation through the eyes of the included other (Aron *et al.*, 1991). Empirically, self-expansion is associated with increased well-being, greater perspective-taking ability and an increased sense of social resources (Aron *et al.*, 2013). Losing a self-expanding relationship is associated with the loss of self-concept clarity (Slotter *et al.*, 2010) and reduced self-concept complexity (Lewandowski *et al.*, 2006).

As it relates to the inclusion of mother or grandmother in the self, we take this conceptualization of what self-expansion is to imply the following. First, when children include their mother in their sense of self, they include both their mother’s attributes in their sense of self and their mothers’ perspective on who they are. Second, when their mothers leave, this aspect of LB children’s sense of self gets torn, undermining their clarity about who they are. Third, that LB children may cope by including their grandmother in their sense of self. We expect that this replacement can help LB children regain a sense of resources and provide them with a positive perspective on themselves. For example, regarding resources, LB children might (implicitly) experience the sense that ‘My grandmother has the will to keep going even when she is tired, I have that will too’. Regarding experiencing a positive perspective, LB children might (implicitly) experience the sense that ‘My grandmother expects me to behave myself and study hard, and since we are connected, it is *my* goal to behave well and study hard’.

## Operationalizing and assessing self-expansion

Self-expansion is typically operationalized using Aron *et al.*’s (1992) Inclusion of Others in Self (IOS). The IOS is a single-item scale successfully implemented cross-culturally (e.g. Tan *et al.*, 2015) and found reliable in use with Chinese elementary school and college student participants (e.g. Feng *et al.*, 2014). The IOS scale presents seven pairs of labeled circles. The labeled pairs are ordered such that the first pair does not overlap and the last pair fully overlaps. Participants select the labeled pair of circles that best represents their relationship with a particular other person. Each circle in a pair is labeled with one circle labeled ‘me’ and the other circle labeled a particular other person (e.g. grandmother, mother).

## Function: cognitive consequences of self-expansion

Having conceptualized self-expansion and operationalized it as choosing an overlapping circle pair to represent felt connection to another person, we consider the prediction that self-expansion affects cognition. To test this prediction, we capitalize on the ‘self-referent’ effect, which refers to average enhanced recall of information incidentally related to the self rather than to something else or nothing (Klein *et al.*, 1989).^1^ Effects are developmentally robust: higher accuracy for incidentally self-linked information is found among participants as young as 4 (Zhou *et al.*, 2010; Cunningham *et al.*, 2014) or 5 years of age (Gutchess *et al.*, 2007). This memory boost is due, in part, to the automatic elaboration of information that is associated with the self (Rogers *et al.*, 1977; Symons and Johnson, 1997). Given this reasoning, a consequence of self-expansion should be enhanced incidental recall of information linked to the included other through co-activation in memory of self-and-close-other associative knowledge networks (Smith *et al.*, 1999). Indeed, self-referent effects are smaller when the comparison is to recall of information incidentally linked to a person one feels close to (e.g. mother, Zhu *et al.*, 2007; a romantic partner, Zhou and Su, 2008). Hence, we predict that if LB children are more likely to include their grandmother in their self than other children, they should have better incidental recall for grandmother-linked information than other, NLB, children.

## Physiological markers: neurological consequence of self-expansion

Having considered effects on cognition, in this section, we consider the prediction that self-expansion is reflected at the neurophysiological level. To do so, we focus on neurological signatures at the level of ERPs, an electrophysiological real-time non-invasive measure of responsivity to information. Specifically, we focus on the ERP component labeled P3, which is associated with attentional processing, working memory processing, decision-making and response preparation (Nieuwenhuis *et al.*, 2005; Polich, 2007). P3 amplitude is proportional to the amount of attentional resources engaged in processing a given stimulus (Johnson, 1986) and is not influenced by factors related to response selection or execution (Crites *et al.*, 1995). Self-related processing is usually reflected in an increase in the P3 amplitude (Gray *et al.*, 2004; Tacikowski and Nowicka, 2010, for reviews, Knyazev, 2013; Fan *et al.*, 2016). To the extent that being LB affects self-expansion to include grandmother in the self, then processing for grandmother should lead to increased attention, detectable as enhanced P3 responses in memory tasks used to measure self-referent effects. We test the prediction that LB children will show greater P3 responses when processing grandmother-related information than NLB children.

## Current studies

We tested our prediction that LB children (Study 1) and young adults who grew up LB (Studies 2 and 3) would include grandmother in their sense of self by comparing IOS scores for mother and grandmother, the memory boost participants receive from linking information to self, and in Study 3, P3 amplitude response to this memory task.

### Human subjects

We obtained approval from the Institutional Review Board (IRB) of the Faculty of Psychology, Southwest University, prior to data collection. Study 1 was conducted at a middle school, so we also obtained approval for the procedure and protocol from the middle school Research Review Board. Middle school students were not reimbursed, but we reimbursed college students the equivalent of $1.6 (Study 2) or $8 (Study 3, which included the ERP). Throughout, ethical standards were in line with the Helsinki Declaration and its later amendments or comparable ethical standards.

### Sample size and power

To determine sample size, we used the meta-analytic average effect size in self-reference studies (Symons and Johnson, 1997, *d* = 0.50) to calculate our planned sample size to attain power = 0.80, and alpha = 0.05. This a priori power analysis yielded a sample size of 66 for the within-between interaction between task and LB-status. Actual sample size varied somewhat due to limitations based on classroom size (Study 1) and missing data on the screening questionnaire (Study 2).

### Covariates

We explored gender and site effects, including them as controls in analysis if they were related to the dependent variable as detailed next and in Supplementary Materials.

### Dependent measures

We report results for each dependent measure separately as each is distinct, as detailed in Supplementary Materials.

### Study set-up

In each study, participants worked individually on a computer running E-Prime software. Text was black and screen background was white.

## Study 1

### Sample

Students (*N* = 66) from a rural middle school near Chongqing, a city of 30 million in Southwest China, participated in an ‘incidental memory’ study. About half of students were not left behind (NLB, *n* = 34, 38.2% males, *M*_age_ = 12.76, s.d. = 0.78), lived with their parents separately from their grandparents. The other half of students were LB (*n* = 32, 53.1% male, *M*_age_ = 12.84, s.d. = 0.92), lived without their parents and with their grandmothers as main caregivers. LB children were about 3 years old (*M* = 3.84, s.d. = 1.80, range 1–7) when their parents left to work in cities.

### Procedure

#### Pilot

Prior to the main study, we pilot-tested 56 trait adjectives for valence (1 = negative, 5 = positive) and familiarity (1 = not at all familiar, 5 = extremely familiar) in a different group (*N* = 45, *M*_age_ = 13.18, s.d. = 1.11) of LB (*n* = 22) and NLB (*n* = 23) students from the same school. Adjectives came from the *Common Classified Adjective Chinese Dictionary* (Fu, 2010). LB and NLB children did not differ on their ratings (valence *F*(1,43) = 0.33, *P* = 0.566; familiarity *F*(1,43) = 0.59, *P* = 0.448).

#### Procedure

We constructed our recall task by randomly dividing the 56 piloted adjectives into four sets of 14, equating on valence (*F*(3,41) = 0.23, *P* = 0.873) and familiarity (*F*(3,41) = 0.03, *P* = 0.993). The words randomly assigned to each target are presented in Supplementary Table S1.

Students were given five practice trials to be sure they understood the instructions: ‘Does the word describe you?’ (‘*ziji*’), ‘Does the word describe your mother?’ (‘*mama*’), ‘Does the word describe your grandmother’ (‘*nainai’*) or ‘Is this a good word?’ (control condition). We balanced the order in which students responded to each of the targets (you ‘*ziji*’, mother ‘*mama*’, grandmother ‘*mama*’, good word) across participants by using a Latin Square design. The instruction was presented (3 seconds) followed by a target screen (4 seconds) showing the target (top of the screen), one of the 56 trait adjectives (middle of screen), and the instruction: press F-key for ‘yes’, press J-Key for ‘no’ (bottom of screen). Each target screen was followed by a 500 ms pause. After responding ‘yes’ or ‘no’ to each of the 56 trait adjectives, students completed a 2-minute arithmetic filler task, followed by a surprise recall task in which they were to type in all the adjectives they could recall. After the recall task, students completed the two IOS scales (Aron *et al.*, 1992) in randomized order. In one IOS, they saw circles labeled ‘me’ and ‘mother’ and rated how close they felt to their mother. In the other IOS, they saw circles labeled ‘me’ and ‘grandmother’ and rated how close they felt to their grandmother. The IOS Chinese labeling is presented in Supplementary Materials. Finally, students reported their age, gender, living arrangement, if they were LB, and if so, how old they were when they were first LB.

### Results

#### LB children include grandmother in the self

IOS responses supported our predictions regarding grandmother and mother self-expansion. LB children were more likely than NLB children to self-expand to include their grandmother in their self-concept, while NLB children were more likely than LB children to self-expand to include their mother in their self-concept. Specifically, a mixed-design analysis of variance (ANOVA) with LB-status as our between-subjects factor and target (mother, grandmother) as our within-subjects factor revealed an LB-status by target interaction (*F*(1,64)  16.48, *P <* 0.001, ${\eta}_{\mathrm{p}}^2$ = 0.205), which moderated the main effects of LB-status (*F*(1,64) = 1.18, *P* = 0.281, ${\eta}_{\mathrm{p}}^2$ = 0.018) and target (*F*(1,64) < 0.01, *P* = 0.985, ${\eta}_{\mathrm{p}}^2$ < 0.001). Sensitivity power analysis shows that the minimum detectable effect size (*n* = 66, power of 0.80, *r*_within_ = −0.07, and *P* = 0.05) for within-between interaction is *f* = 0.26. Hence, our study was sufficiently powered to detect the interaction between target and LB-status (*f* = 0.51).

**Fig. 1:**
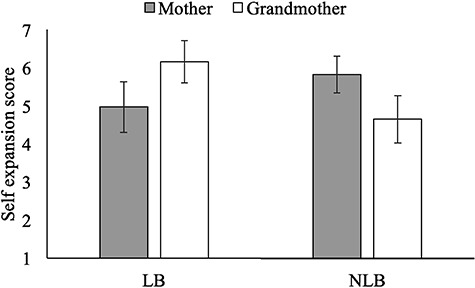
Study 1: Overlap of mother and grandmother with self as function of LB-status*.* Error bars represent 95% CI.

We decomposed this interaction. First, we examined the effect of LB-status on self-expansion to include grandmother, finding that LB children included grandmother in self more than NLB children (*F*(1,64) = 12.65, *P* = 0.001, *d* = 0.50). Second, we examined the effect of LB-status on self-expansion to include mother, finding that mother was included more among NLB children than LB children (*F*(1,64) = 4.28, *P* = 0.042, *d* = 0.51). Effects are presented graphically in Figure 1 and descriptively in Table 1.

**Table 1:** Study 1: Mean (s.d.) self-mother and self-grandmother overlap scores of LB and NLB students (1 = no overlap to 7 = complete overlap)

LB-status	Overlap between self and …		Row effect size *d*
	Mother	Grandmother	
LB	4.97 (1.91)	6.16 (1.59)	0.45
NLB	5.82 (1.42)	4.65 (1.84)	0.58
Column effect size *d*	0.51	0.88	

*Note*: *d* = Cohen’s *d*. We computed row effects sizes using pooled standard deviations and a repeated measures design.

#### LB children show superior grandmother-related recall

**Fig. 2:**
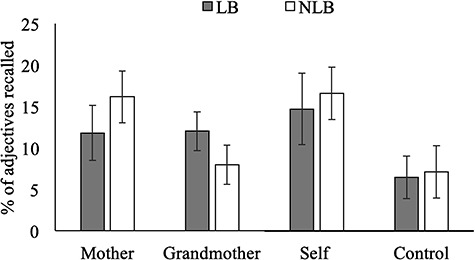
Study 1: Memory for words encoded in reference to mother, grandmother, self, and valence among students (LB, NLB). Error bars represent 95% CI.

Using incidental recall, we found support for our prediction that self-expansion has functional consequences. LB children had better recall for information incidentally linked to grandmother than did NLB children. After clarifying comparability (LB and NLB children did not differ in overall recall, *F*(1,64) = 0.29, *P* = 0.594, ${\eta}_{\mathrm{p}}^2$ = 0.002; recall of self-related, *F*(1,64) = 0.49, *P* = 0.49 or valence-related, *F*(1,64) = 0.11, *P* = 0.74 adjectives), we conducted a mixed-design ANOVA on recall. Encoding condition (mother, grandmother) was the within-subject factor and LB-status was the between-subject factor. We found a main effect of condition (*F*(1,64) = 8.05, *P* = 0.006, ${\eta}_{\mathrm{p}}^2$ = 0.11) and the predicted condition by LB-status interaction (*F*(1,64) = 8.97, *P* = 0.004, ${\eta}_{\mathrm{p}}^2$ = 0.12). These effects are detailed in Table 2 and shown graphically in Figure 2. Since the minimum detectable effect size is *f* = 0.25, we were sufficiently powered to detect the condition by LB-status interaction (*f* = 0.37).

**Table 2:** Study 1: LB and NLB children’s mean (s.d.) percentage recall for words incidentally linked to self, to mother, to grandmother and to word valence (control condition)

LB-status	Mean percentage of recall by encoding condition				
	Mother	Grandmother	Self	Control	${\eta}_{\mathrm{p}}^2$
LB	11.83% (9.70)	12.05% (6.89)	14.73% (12.43)	6.47% (7.33)	0.16
NLB	16.18% (9.52)	7.98% (6.75)	16.60% (9.09)	7.14% (8.96)	0.27
Cohen’s *d*	0.45	0.60	0.17	0.08	

*Note:* Participants were middle school students. In each condition, a perfect score of 100 (100%) would be obtained if the participant correctly remembered each word presented with each figure in the first phase of the experiment.

We decomposed this interaction. First, we examined the effect of LB-status in the grandmother-encoding condition. As predicted, LB children recalled more grandmother-related adjectives than NLB children (*F*(1,64) = 5.87, *P* = 0.02). Second, we examined the effect of LB-status in the mother-encoding condition, finding a non-significant reversal, NLB children recalled more mother-related adjectives than LB children (*F*(1,64) = 3.37, *P* = 0.071). Results should be interpreted in light of the self-reference effect. Both LB and NLB groups showed a self-reference effect (LB group: *t*(31) = 3.57, *P* = 0.001; NLB group: *t*(33) = 4.45, *P* < 0.001). Both groups also showed a mother-reference effect (LB group: *t*(31) = 3.10, *P* = 0.004; NLB group: *t*(33) = 3.83, *P* = 0.001), such that their recall of self- and mother-related adjectives was better than recall of adjectives in the control condition. Only LB children (*t*(31) = 4.39, *P* < 0.001), not NLB children (*t*(33) = 0.47, *P* = 0.64), demonstrated grandmother-reference effect.

## Study 2

### Sample

Subject pool undergraduates (*N* = 128) at Southwest University (*n* = 58) and Chongqing University of Arts and Sciences (*n* = 70) living on the college campuses were recruited for a study on memories of family life experiences’. We used a pre-college family life prescreen to attain formerly LB participants (reported an LB life experience and lived with a grandmother) and NLB participants (reported always living with parents in households that did not include grandparents). On closer inspection, 11 prescreen questionnaires were ambiguous regarding family life experience, making it impossible to assign them to LB or never left-behind as children (NLB), yielding a final sample of *N* = 117 (NLB: *n* = 61, 21.3% males, *M* = 20.1, s.d. = 2.04; Formerly LB: *n* = 56, 12.5% male, *M*_age_ = 19.6, s.d. = 1.79). On average, formerly LB students were left behind at the age of 6 (*M =* 6.36, s.d. = 3.64, range 1 to 14) in the care of a grandmother for more than 7 years (*M* = 7.57, s.d. = 4.46, range 1–18 years). We did not prescreen for gender, but our sample over-represented females, especially in the formerly LB condition.

**Fig. 3:**
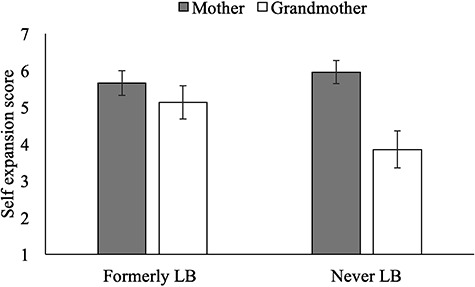
Study 2: Inclusion of mother and grandmother in self as function of former LB-status. Error bars represent 95% CI.

### Procedure

We used the same tasks and procedures as in Study 1, making two changes in our adjective task. First, we changed the control condition instruction from asking for adjective valence to asking if each adjective described a well-known literary celebrity named *Luxun*. Second, we increased the number of adjectives presented in each condition from 14 to 20. We piloted our 80 trait adjectives, taken from the *Common Classified Adjective Chinese Dictionary* (Fu, 2010) for valence (1 = negative, 5 = positive) and familiarity (1 = not familiar at all, 5 = extremely familiar) in a separate sample of Southwest University undergraduates (*n* = 18). Adjectives were randomly assigned to four sets so that neither valence (*F*(3,15) = 0.02, *P* = 0.996) nor familiarity (*F*(3,15) = 1.28, *P* = 0.316) differed by set and each set included 20 adjectives (Supplementary Table S2).

### Results

#### Preliminary descriptive analyses

As detailed in our Supplementary Materials, gender affected IOS scores (not recall) and site affected recall (not IOS); hence, we included gender as a covariate in IOS analyses and site as a covariate in recall analyses.

#### Formerly LB undergraduates include grandmother in the self

As can be seen in Figure 3 graphically, formerly LB undergraduates (*M* = 5.13, s.d. = 1.75) included grandmother in self more than NLB undergraduates (*M* = 3.85, s.d. = 2.06). The groups did not differ in inclusion of mother in self (*M*_formerly LB_ = 5.66, s.d. = 1.27, *M*_NLB_ = 5.95, s.d. = 1.28). As detailed in Table 3, a mixed-design ANOVA controlling for gender yielded the predicted LB-status by target interaction, *F*(1,113) = 12.51, *P <* 0.001, ${\eta}_{\mathrm{p}}^2$ = 0.12, which moderated LB-status (*F*(1,113) = 7.30, *P* = 0.008, ${\eta}_{\mathrm{p}}^2$ = 0.06) and target (*F*(1,113) = 9.41, *P* = 0.003, ${\eta}_{\mathrm{p}}^2$ = 0.08) main effects.

**Table 3:** Study 2: Self-expansion to include mother and grandmother: mean (s.d.) self-mother and self-grandmother overlap scores of formerly LB and NLB students

LB-status	Overlap between self and …		Row effect size *d*
	Mother	Grandmother	
Formerly LB	5.66 (1.27)	5.13 (1.75)	0.25
NLB	5.95 (1.28)	3.85 (2.06)	1.34
Column effect size *d*	0.23	0.67	

*Note*: Participants were undergraduates who had (formerly LB) or had not (NLB) been LB as children. Overlap scores range from 1 = no overlap, 7 = complete overlap between self and other. *d* = Cohen’s *d*. We computed row effect sizes using pooled standard deviations and a repeated measures design.

We decomposed the interaction, finding that formerly LB students had higher grandmother-in-self scores than NLB students (*F*(1,115) = 12.89, *P <* 0.001, *d* = 0.67) and NLB students had non-significantly higher mother-in-self scores than formerly LB students (*F*(1,115) = 1.51, *P* = 0.22, *d* = 0.23). Sensitivity power analysis shows that the minimum detectable effect size (*n* = 117, power of 0.80, *P* = 0.05) for within-between interaction is *f* = 0.17. Hence, our study was sufficiently powered to detect the LB-status by target interaction (*f* = 0.37).

#### Former LB status does not affect incidental recall

**Fig. 4:**
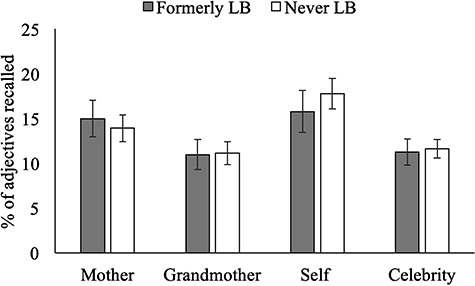
Study 2: Memory for words encoded in reference to mother, grandmother, self, and celebrity control by LB status. Error bars represent 95% CI.

We conducted a mixed-design ANOVA on percentage of adjectives recalled, with encoding target (mother, grandmother) as the within-subject factor and LB-status as the between-subject factor, controlling for site. LB-status did not affect recall overall (*F*(1,113) = 0.28, *P* = 0.60) and did not interact with target (*F*(1,113) = 0.55, *P* = 0.46) as detailed in Table 4 and displayed graphically in Figure 4. Students recalled adjectives linked to self (*t*(116) = 5.32, *P* < 0.001) and mother (*t*(116) = 3.06, *P* = 0.003) more than adjectives linked to the celebrity control. They were just as likely to recall adjectives linked to grandmother or celebrity control (*t*(116) = −0.45, *P* = 0.65).

**Table 4:** Study 2: Formerly LB and never left-behind (NLB) undergraduate’s mean (s.d.) Percentage recall for words incidentally linked to self, to mother, to grandmother and to Luxun (famous author which constituted our control condition)

LB-status	Mean percentage of recall by condition				
	Mother	Grandmother	Self	Control	${\eta}_{\mathrm{p}}^2$
Formerly LB	15.00% (8.69)	10.98% (7.35)	15.80% (9.57)	11.25% (5.97)	0.16
NLB	13.93% (8.12)	11.15% (6.61)	17.79% (9.33)	11.64% (5.75)	0.27
Cohen’s *d*	0.13	0.02	0.21	0.07	

*Note:* Participants were undergraduates who had (formerly LB) or had no (NLB) been LB in their school years. In each condition, a perfect score of 100 (100%) would be obtained if the participant correctly remembered each word presented with each figure in the first phase of the experiment.

## Study 3

### Sample

Participants were Southwest University undergraduates (*N* = 45, LB *n* = 22, 50% male, *M*_age_ = 20.04, s.d. = 1.21; NLB *n* = 23, 39.1% male, *M*_age_ = 21.22, s.d. = 1.73). We defined LB as having experienced an absence of both parents and instead living with grandparents with grandmother as the main caregiver during kindergarten and/or primary school (Zhang *et al.*, 2011). We screened for right-handedness and normal or corrected-to-normal vision without neurological conditions for the ERP task. We recruited participants as we did in Study 2. However, due to researcher’s error, the length of time that students were LB and the year they started being LB were not recorded.

### Procedure

We used Adobe Photoshop CS3 to produce images of each adjective. When participants came to the lab, we outfitted them with a *BrainProducts* elastic cap with tin electrodes in 64 scalp positions set up according to the international 10–20 system. We followed standard procedure; specifically, we used the electrode at the right mastoid as a reference and the one on the medial frontal aspect as a ground electrode. We recorded vertical electrooculograms (EOGs) supra and infraorbitally at the left eye and horizontal EOGs at the left and right orbital rim. EEG and EOG activity were amplified with a DC ~100 Hz bandpass and continuously sampled at 500 Hz. All electrode impedances were maintained below 5 KΩ.

Having been set up, participants first completed Tulving’s (1985) version of the incidental recall task (the remember/know recognition task) using the procedure and adjective list detailed in Yang and Huang (2007). Participants indicated (Yes/No) if each adjective could be used to describe a target (e.g. self) and considered all adjectives connected to a target before moving to the next target. As in Study 2, the targets were oneself (*ziji*), one’s mother (*mama*), one’s grandmother (*nainai*) or Luxun (a famous Chinese writer), presented in randomized order. Participants completed the Tulving task (including 10 practice trials, a filler and surprise recall), three IOS scales (mother, grandmother and celebrity) in randomized order and finally the demographics questions. We collected ERP data during the Yes/No describes target part of the Tulving task.

In the Tulving task, each adjective was presented on the screen for 3 seconds followed by a fixation point (+), shown for 600–1000 ms. After completing the Yes/No describes target part of the Tulving task, students completed a 5-minute 9 × 9 Sudoku filler task. Then, they were given a surprise recall task. In this task, they were shown 200 adjectives, which they had seen before randomly intermixed with 100 equally familiar similarly valenced but new adjectives. Each adjective was presented on the screen for 4 seconds followed by a fixation point (+), shown for 600–1000 ms. Students were told to press the ‘1’ key if the word was new, to press the ‘2’ key if they recognized the word (*knew*) but could not remember the details (K response) and to press the ‘3’ key if they *remembered* the details of where the word had appeared (R response). We only used the ‘remember’ scores following van den Bos *et al.* (2010) who found that self-referent processing enhances recollection (‘remember’) but not recognition (‘know’).

Practice trials were dropped from analyses. Following Yang and Huang (2007), each target was linked to 62 adjectives at stage one, but the first set of six adjectives and a last set of six adjectives were not considered in the surprise recall task to reduce primacy and recency effects on memory. We obtained event-locked ERPs by averaging ERP responses for each target during the describe Yes/No task (averaging epochs began 200 ms prior to stimulus onset and ended 1200 ms after stimulus onset). We analyzed average amplitude of P3 (340–600 ms), focusing on the central–parietal sites (CPz and Pz) scalp regions in which it has maximum amplitude (Tacikowski and Nowicka, 2010; Auerbach *et al.*, 2016). We excluded trials contaminated by eye blinks (vertical electrooculographic, VEOG exceeding ±100 μV relative to baseline) or other artifacts (a voltage exceeding ±50 μV at any electrode location relative to baseline).

### Results

#### Being LB influences inclusion of grandmother and mother in the self

We used mixed-design ANOVA with LB-status as the between-subject variable and target as the within-subject variable. As detailed in Table 5, we found the predicted LB-status by target interaction (*F*(1, 43) = 14.34, *P* < 0.001, ${\eta}_{\mathrm{p}}^2$ = 0.250), a non-significant main effect of LB-status (*F*(1, 43) = 0.02, *P* = 0.895, ${\eta}_{\mathrm{p}}^2$ < 0.001) and a significant main effect of target, *F*(1, 43) = 8.99, *P* = 0.004, ${\eta}_{\mathrm{p}}^2$ = 0.173. Our sensitivity power analysis showed that the minimum detectable effect size (*n* = 45, power of 0.80 and *P* = 0.05) for within-between interaction is *f* = 0.25, so our study was sufficiently powered to detect this interaction (*f* = 0.58).

**Table 5:** Study 3: Formerly LB and never left-behind (NLB) students: overlap between self and mother, between self and grandmother and between self and Luxun (celebrity author, constituted our control condition)

LB-status	Overlap between self and …			Difference in self-mother and self-grandmother overlap effect size *d*
	Mother	Grandmother	Celebrity	
Formerly LB	5.64 (.58)	5.73 (.83)	1.41 (.50)	0.14
NLB	6.04 (.93)	5.26 (.96)	1.30 (.63)	0.90
Column effect size *d*	0.52	0.51	0.19	

*Note*: Participants were undergraduates who had (formerly LB) or had not (NLB) been LB as children. Self-other overlap scores range from 1 = no overlap to 7 = complete overlap. *d* = Cohen’s *d*.

**Fig. 5:**
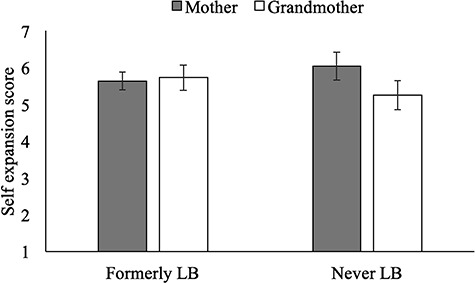
Study 3: Inclusion of mother and grandmother in one’s self as function of former LB status. Error bars represent 95% CI.

We decomposed the interaction by comparing the inclusion of mother and grandmother in self scores of formerly LB and NLB students. As displayed graphically in Figure 5, LB students had higher grandmother-in-self scores (*M* = 5.73, s.d. = 0.83) than NLB students (*M* = 5.26, s.d. = 0.96), *F*(1,43) = 3.02, *P* = 0.09, *d* = 0.52. In contrast, NLB students had higher mother-in-self scores (*M* = 6.04, s.d. = 0.93) than LB students (*M* = 5.64, s.d. = 0.58), *F*(1,43) = 3.08, *P* = 0.09, *d* = 0.52. Results, though medium-sized, were not significant given our small sample size. We also had an include-celebrity-in-self IOS to compare to. In support of the validity of our finding of LB and NLB differences in including grandmother and mother in self, LB and NLB students did not differ in their celebrity-in-self scores (*F*(1,43) = 0.37, *P* = 0.544). They included the celebrity in self to a much lower degree than mother and grandmother (*P*s < 0.001).

#### Formerly LB undergraduates show superior cognitive processing for grandmother

We focused on the more diagnostic percentage of correct R (remember) responses for self-, mother-, grandmother- and celebrity-related adjectives as our dependent measures (less diagnostic K responses are detailed in Supplementary Materials). To directly test our self-expansion hypothesis, we conducted a mixed-design ANOVA on percentage of correct remember responses, with condition (mother, grandmother) as the within-subject factor and LB-status as the between-subject factor. As detailed in Table 6, we found a main effect of condition on R recall (*F*(1,43) = 13.28, *P* = 0.001, ${\eta}_{\mathrm{p}}^2$ = 0.24) and the predicted interaction between condition and LB-status (*F*(1, 43) = 8.34, *P* = 0.006, ${\eta}_{\mathrm{p}}^2$ *=* 0.16). Since the minimum detectable effect size is *f* = 0.12 (*n* = 45, power of 0.80, *r*_within_ = 0.85, and *P* = 0.05), our study was sufficiently powered to detect this task by LB-status interaction (*f* = 0.44).

**Table 6:** Study 3: Formerly LB and never left-behind (NLB) undergraduate’s mean (s.d.) percentage remember score for words incidentally linked to self, to mother, to grandmother and to Luxun (famous author)

LB-status	Mean percentage of remember responses (s.d.)				Row effect size *d* for mother grandmother difference
	Mother	Grandmother	Self	Celebrity	
Former LB	40.69 (19.15)	50.20 (18.44)	56.68 (22.11)	39.24 (20.01)	1.19
NLB	42.91 (19.69)	44.01 (19.41)	52.15 (19.72)	38.38 (18.28)	0.10
Column effect size *d*	0.11	0.33	0.22	0.01	

*Note:* Participants were undergraduates who had ever (formerly LB) or had never (NLB) been LB in their school years. In each condition, a perfect score of 100 (100%) would be obtained if the participant correctly remembered each word presented with each figure in the first phase of the experiment. *d =* Cohen’s *d*. For within-subject mother–grandmother difference, we used the pooled standard deviation to compute effect size.

We decomposed this interaction by examining percentage of remember responses linked to mother and grandmother by LB-status. As predicted by our self-expansion to include grandmother for LB children hypothesis, formerly LB undergraduates were better at remembering adjectives incidentally linked to their grandmother (*M* = 50.20, s.d. = 18.44) than to their mother (*M* = 40.69, s.d. = 19.15, *t*(21) = 5.53, *P* < 0.001, *d* = 1.19). We found no such difference for NLB undergraduates (*t*(22) = 0.47, *P* = 0.64, *d* = 0.10). Moreover, both formerly LB (*t*(21) = 6.75, *P* < 0.001) and NLB (*t*(22) = 4.67, *P* < 0.001) undergraduates showed the expected self-reference effect, better remembering adjectives incidentally linked to the self than adjectives incidentally linked to a celebrity. This suggests that the superior remembering performance related to grandmother among formerly LB undergraduates indeed reflects expanded self-reference processing.

#### Physiology

**Fig. 6:**
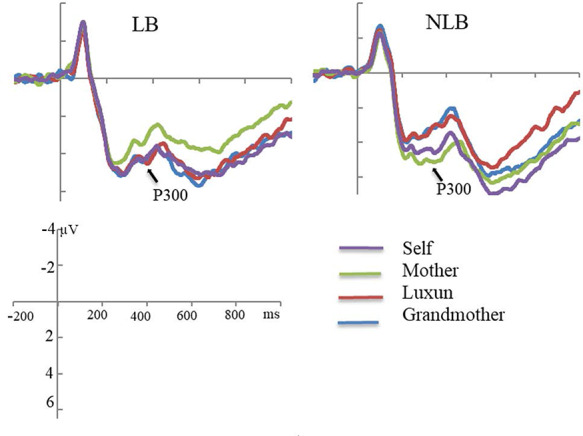
Study 3: Illustration of grand averaged waveforms of electrodes CPz and Pz for self, mother, Luxun, grandmother conditions separately for formerly left-behind (LB, left panel) and never left-behind (NLB, right panel) undergraduates.

We conducted repeated measures ANOVA for P3 amplitude. Figure 6 shows results. We found a significant LB-status by condition interaction (*F*(3, 41) = 5.43, *P* = 0.003, ${\eta}_{\mathrm{p}}^2$ = 0.28) on P3 that moderated the main effect of condition (*F*(3,41) = 1.28, *P* = 0.29, ${\eta}_{\mathrm{p}}^2$ = 0.09) and LB-status (*F*(1, 43) = 0.28, *P* = 0.60, ${\eta}_{\mathrm{p}}^2$ = 0.01). We decomposed the LB by condition interaction (condition effect for NLB students, *F*(3,41) = 4.33, *P* = 0.01, ${\eta}_{\mathrm{p}}^2$ = 0.24; condition effect for LB students *F*(3,41) = 2.42, *P* = 0.08, ${\eta}_{\mathrm{p}}^2$ = 0.15). For NLB students, self and mother evoked larger amplitude than grandmother and celebrity (*Luxun*), *P*s < 0.05, and amplitude did not differ for self and mother (*P* = 0.96) or for grandmother and celebrity (*P* = 0.80). In contrast, for LB students, mother evoked less amplitude than self (*P* = 0.046), grandmother (*P* = 0.01) or celebrity (*P* = 0.03), all other *P*s > 0.05.

Study 3 results replicate our Study 1 findings that being LB affects children’s sense of self. Formerly LB college students included grandmother in their sense of self more than their NLB peers, and this had consequences for their cognitive processing. In Studies 1 and 3, students who were LB remembered words that had been incidentally linked to their grandmother better than words that had been incidentally linked to their mothers. No such effect was found for students who were NLB. We also found evidence for physiological traces of this expanded self to include grandmother, such that formerly LB students had higher amplitude of P3 response for grandmother than for their mother and that this was not the case for their NLB peers.

## Single-paper meta-analysis

We conducted single-paper meta-analyses of Studies 1, 2 and 3 using comprehensive meta-analysis to obtain stable estimates of the effect of LB experience on feeling and functional inclusion of grandmother and mother in the self. Our meta-analytic results are detailed in Tables 7 and 8 and summarized next. First with regards to feeling, our meta-analysis (Table 7) revealed a large-sized difference between LB and NLB participants in how much they included their grandmothers in their sense of self (*d* = 0.70, 95% confidence intervals [CI] = [0.43, 0.97], *Z* = 5.11, *P* < 0.001) and showed that effect sizes did not vary across studies (*Q*(2) = 0.87, *P* = 0.65). LB students, students who are currently or have previously been LB by their parents, include their grandmother in sense of self more than NLB students, students who are currently or were formerly LB. With regard to mothers, our meta-analyses revealed a small-to-moderate significant difference between LB and NLB students in how much they included their mothers in their sense of self (*d* = 0.36, 95% CI = [0.10, 0.63], *Z* = 2.72, *P* = 0.007) and showed that effect sizes do not vary across studies (*Q*(2) = 1.11, *P* = 0.57). LB students included their mother in their sense of self less than NLB students. Second, with regards to function, our meta-analysis (Table 8) revealed a small non-significant (*d* = 0.22, 95% CI = [−.04, 0.48], *Z* = 1.65, *P* = 0.10) increase in automatic processing of information incidentally related to grandmother among LB students, relative to NLB children; this effect was not dependent on study (or measure). We found no difference between LB and NLB students in processing of information incidentally related to mother (*d* = 0.09, 95% CI = [−0.18, 0.35], *Z* = 0.65, *P* = 0.52), and this did not differ by study or measure.

**Table 7:** Meta-analytic summary of the effect of being LB on feeling that grandmother and mother are part of the self

Study	LB-status^a^	Inclusion of grandmother		Inclusion of mother	
		Cohen’s *d*	95% CI	Cohen’s *d*	95% CI
1	Current	0.88	0.38, 1.39	0.51	0.02, 1.00
2	Former	0.67	0.30, 1.04	0.23	−0.14, 0.59
3	Former	0.52	−0.07, 1.12	0.51	−0.08, 1.11
Average effect size		0.70	0.43, 0.97	0.36	0.10, 0.63
Heterogeneity		*Q*(2) = 0.87, *P* = 0.65, *I*^2^ = 0%		*Q*(2) = 1.11, *P* = 0.57, *I*^2^ = 0%	

^a^Current = currently left behind (compared to not left behind), former = left behind prior to college (compared to never left behind).

**Table 8:** Meta-analytic summary of the effect of being LB on automatic processing of words incidentally associated with grandmother and mother

Study	LB-status^a^	Inclusion of grandmother		Inclusion of mother	
		Cohen’s *d*	95% CI	Cohen’s *d*	95% CI
1	Current	0.60	0.10, 1.09	0.45	−0.03, 0.94
2	Former	−0.02	−0.39, 0.34	−0.13	−0.49, 0.24
3	Former	0.33	−0.26, 0.92	0.11	−0.47, 0.70
Average effect size		0.22	−0.04, 0.48	0.09	−0.18, 0.35
Heterogeneity %		*Q*(2) = 4.11, *P* = 0.13, *I*^2^ = 51.37%		*Q*(2) = 3.50, *P* = 0.17, *I*^2^ = 42.83%	

^a^Current = currently left behind (compared to never left behind), former = left behind prior to college (compared to never left behind).

## General discussion

In many regions of the world, parents leave their children behind, seeking work far from home and sending remittances home to support their children and families. Our review of the growing literature on the LB children of these parents highlights both culture-bound variability and lack of knowledge as to what parental separation does to children’s sense of self. In the current paper, we focus on self-expansion of LB children in China. Being LB is all-too-common in China (Fellmeth *et al.*, 2018; Griffiths *et al.*, 2018). It is associated with stress (Ye and Pan, 2011), depression, anxiety (Wong *et al.*, 2009; Wang *et al.*, 2019b), loneliness (Jia and Tian, 2010; Dai et al., 2017), lower popularity (Wang *et al.*, 2015) and worse physical health (Gao *et al.*, 2010; Huang *et al.*, 2015; Griffiths *et al.*, 2018). Negative consequences are particularly likely for children LB by their mothers (Jampaklay, 2006; Duan and Wu, 2009). But children left in the care of grandparents (most commonly grandmothers) are less at risk of these negative outcomes (Jampaklay, 2006; Duan and Wu, 2009) than those left with other relatives, teachers or alone (Huang *et al.*, 2015). Moreover, children LB with grandparents retain their sense that school success is a possible identity and have strategies to attain this future (Bi and Oyserman, 2015). In the current paper, we examined one way in which LB children may cope, which is to include their caretaking grandmother in their sense of self.

Including psychologically close others in one’s sense of self has two aspects—seeing oneself through the (positive) lens of that close other and including that close other’s (positive) attributes and strengths in one’s self (Aron *et al.*, 2013). We compared currently or formerly LB children and children who had never been LB in their inclusion of mother and grandmother in the self. To do so, we asked respondents to choose from different levels of overlapping circles to represent their connection with mothers and with grandmothers (Studies 1–3). Across studies, the effect of being LB on inclusion of grandmother in self was large and consistent, whether children were currently (Study 1) or formerly (Studies 2 and 3) LB. LB children include their grandmother in their sense of self more than other children and never left-behind children include their mother in their sense of self more than ever LB children.

We tested the effect of including mother and grandmother in the self on automatic processing in two ways—incidental encoding (Studies 1 to 3) and electrophysiological evidence of differential attention (ERP, Study 3). Information incidentally linked to mother was automatically encoded and later better recalled than words related to strangers. Information incidentally linked to grandmother was sometimes automatically encoded and later better recalled by LB than by NLB students. We found significant effects in 2 of our 3 studies. This inconsistency which might imply that the effect of inclusion of grandmother in self on automatic processing is dependent on the measure used as the inconsistency was associated with subtle differences in our measurement strategy. Indeed, a mini-meta analyses across studies reveal that the effect of was small and positive but did not attain significance.

With regard to electrophysiological evidence, we found that formerly LB students showed stronger P3 responses when considering whether words described their grandmother than did never left-behind students. This result is important because P3 is an attention response linked to processing information about the self (Knyazev, 2013). For NLB students, P3 response was greater for mother and self than for grandmother and a famous author. For formerly LB students, P3 response was weaker for mother than for self, grandmother or a famous author. The implication that formerly LB children pay less attention to information incidentally linked to mother than to others is congruent with lower inclusion of mother than grandmother in the self. However, the finding regarding famous author contrasts with the lower inclusion in self of famous author than mother and hence should be taken with a grain of salt. Taken together, our results support the idea that LB children include their caregiving grandmothers in the self to compensate for the absence of mother, and this effect may be long-lasting and continuing into adulthood. Because inclusion of close others is normative and emotionally bolstering, our results suggest that including grandmother in the self may bring a positive impact even when LB children have grown up and left home.

### Self-expansion of LB children

Our results fit with the broader literature on the necessity of strong caregiving bonds. The currently or formerly LB children in our studies had been physically deprived of their parents in their early lives and provided with grandmothers instead. Including caregiving grandmother as a substitute for mother in one’s sense of self fits evolutionary and culturally shaped scripts (Tan *et al.*, 2015). It allows LB children to maintain an intimate relationship with their caregiver, facilitating emotional bonding (Zhang and Fuligni, 2006) and substituting for a disrupted parent–child cohesive bond (Zhao *et al.*, 2015).

Globally, grandparents serve as surrogate parents to their grandchildren (Dolbin-MacNab and Yancura, 2018) with cultural collectivism being associated with more positive response to taking this role (Hayslip *et al.*, 2017). In China, in particular, being a grandparent is an important social role for older adults (Wang *et al.*, 2019a). In traditional Chinese culture, grandparents often care for their grandchildren, and grandchildren in turn are expected to respect and care for their grandparents when they grow up (Xu *et al.*, 2018). Children fare better when they are left with grandparents than with other caretakers (e.g. other relatives, teachers, Huang *et al.*, 2015). The implication, which we did not measure, is that effects are reciprocal. That is, grandparents in our sample may have self-expanded to include their grandchildren in their sense of self just as their grandchildren self-expanded to include at least grandmother in their sense of self. Positive impact could accrue to both grandparent and child well-being.

### Limitations

A strength of our studies is that we focused on an important but understudied group that is growing in size—currently and formerly LB children and that we focused on a coping resource—including grandmothers in sense of self. However, like all studies our studies have limitations. We address four of these here. First, normal development entails shift in mental models of self, of parents and of the parent–child bond (Coleman, 2001). It is possible that as formerly LB children enter adulthood, they reframe their bond with their mother and grandmother to shift toward the culturally normative model of including mother, rather than grandmother in their sense of self. The cross-sectional nature of our studies means that we cannot directly address whether such change over time occurs. We do not have data on whether grandmothers are still alive or how much contact students currently have with their grandmothers or mothers. Instead, we provide three snapshots of children experiencing being left behind and of students who were left behind when they were children. We infer from these that emotional bonding is stable over time (self-report and physiological measures), whereas cognitively, grandmother’s potency may fade (effects are variable in the formerly left behind group). Second, our results may be dependent on the ways in which parental migration unfolds in cultural context. Chinese adolescents understand parental migration in terms of benefits to family (Dai and Chu, 2018; Fu and Law, 2018). Though China is a large country and is worthy of our sole attention, our studies are limited in that we cannot know if the findings generalize to other countries or cultural frames. Third, our measures highlight an important aspect of self-concept—inclusion of close others in the self. Thus, our studies cannot address self-functions, such as effectiveness in self-regulation when grandmother and mother bonds are on the mind. A promising avenue for future research would be to investigate how self-inclusion of close others can impact self-regulation in service of emotional health and academic attainment. Fourth, we focus on mother and grandmother as the culturally most common and central caregiving adults. It is possible that our results generalize to any central caregiving adults, but our data cannot address this possibility. These interesting next steps await future research.

## Conclusions and future directions

We document grandmother’s enduring compensatory power for children who are or were LB by their parents who migrated for economic reasons and left their very young children to be raised in their typically rural hometowns by a caregiving grandmother. Effects are visible in self-report of emotional connection and in physiological responses and are smaller and less consistent when a different indirect measure (incidental recall) is used. Our results point to ways to study coping among children stressed by loss of parental presence and highlight the need to understand what inclusion of grandmother in sense of self can do for these children. Given the very large and increasing number of children affected by parental migration, future research on this topic is sorely needed and should expand to documenting the process by which including caregivers in sense of self bolsters children’s emotional and motivational resilience.

## Supplementary Material

scan-19-396-Supplementary_Material_nsaa062

## Footnotes

1 Self-referent scores are obtained by comparing the accuracy of recall a person has to information randomly linked at encoding to the self or to something else.
