# Supplementary material for: Left behind, not alone: feeling, function and neurophysiological markers of self-expansion among left-behind children and not left-behind peers
Source: Soc Cogn Affect Neurosci. 2020 May 4;15(4):467–78. doi: 10.1093/scan/nsaa062 (PMC7308663; doi:10.1093/scan/nsaa062)
Supplement: scan-19-396-Supplementary_Material_nsaa062 [file scan-19-396-supplementary_material_nsaa062.docx]

**LEFT BEHIND BUT NOT ALONE SUPPLEMENTAL MATERIALS**

**Measures**

**Studies 1 to 3: Chinese language labelling inclusion-of-other-in-self**

1. Inclusion of Self and Mother

**1、请在下面的图形中选择最符合你和你母亲关系的图形。**

自己

母亲

**1**

自己

母亲

**2**

自己

母亲

**3**

自己

母亲

**4**

自己

母亲

**7**

自己

母亲

**6**

自己

母亲

**5**

2. Inclusion of Self and Grandmother

**2、请在下面的图形中选择最符合你和你的奶奶关系的图形。**

自己

奶奶

**1**

自己

奶奶

**2**

自己

奶奶

**3**

自己

奶奶

**4**

自己

奶奶

**7**

自己

奶奶

**6**

自己

奶奶

**5**

**Studies 1 to 3: Word List Used in the Recall Task by Study**

Table S1 *Study 1: Word List by Target Condition*

| Self | | Mother | | Grandmother | | Word | |
| --- | --- | --- | --- | --- | --- | --- | --- |
| 刻苦 | assiduous | 体贴 | considerate | 积极 | active | 幽默 | humorous |
| 真诚 | sincere | 坦诚 | frank | 优秀 | excellent | 温和 | gentle |
| 开朗 | cheerful | 善良 | kind | 友好 | friendly | 乐观 | optimistic |
| 诚实 | honest | 大方 | generous | 认真 | serious | 亲切 | cordial |
| 努力 | effortful | 聪明 | smart | 负责 | responsible | 机灵 | clever |
| 勇敢 | brave | 坚强 | strong | 细心 | careful | 和蔼 | nice |
| 耐心 | patient | 高尚 | noble | 随和 | easygoing | 能干 | competent |
| 讨厌 | nasty | 严厉 | severe | 幼稚 | naive | 固执 | stubborn |
| 小气 | stingy | 暴躁 | fierce | 胆小 | coward | 偏心 | eccentric |
| 冷淡 | cold | 自私 | selfish | 悲观 | pessimistic | 任性 | headstrong |
| 呆板 | rigid | 可恨 | hateful | 狡猾 | crafty | 缺德 | wicked |
| 邪恶 | evil | 无能 | incompetent | 狠心 | heartless | 刻薄 | harsh |
| 贪心 | greedy | 奸诈 | treacherous | 卑鄙 | despicable | 消极 | negative |
| 虚伪 | hypocritical | 软弱 | weak | 骄傲 | proud | 糊涂 | silly |

*Note*: Self *(Does This Describe You?),* Mother *(Does This Describe Your Mother?),* Grandmother *(Does This Describe Your Grandmother?),* Word *(Is This a Positive Word?)* Students could respond Yes or No to each word. Condition order was balanced across students by using a Latin Square design.

Table S2 *Study 2*: *Word List by Target Condition*

| Self | | Mother | | Grandmother | | Luxun | |
| --- | --- | --- | --- | --- | --- | --- | --- |
| 愉快 | happy | 勇敢 | brave | 可耻 | shameful | 坦率 | frank |
| 堕落 | degenerate | 慷慨 | generous | 野蛮 | brutal | 虚荣 | vanity |
| 放纵 | indulgent | 残忍 | brute | 聪明 | smart | 可爱 | cute |
| 轻率 | thoughtless | 仔细 | careful | 豪迈 | heroic | 谨慎 | cautious |
| 肮脏 | dirty | 顽强 | tenacious | 真挚 | sincere | 狂妄 | arrogant |
| 果断 | decisive | 风趣 | funny | 小气 | stingy | 亲切 | cordial |
| 节约 | saving | 无情 | merciless | 庸俗 | vulgar | 荒唐 | absurd |
| 英明 | wise | 阴险 | insidious | 忠厚 | honest | 下流 | dirty |
| 正直 | integrity | 凶狠 | fierce | 卓越 | excellent | 胆小 | coward |
| 仁义 | righteousness | 拖拉 | tardy | 专横 | imperious | 大方 | generous |
| 盲目 | blind | 谦虚 | modest | 认真 | serious | 偏心 | eccentric |
| 幼稚 | naïve | 狡猾 | tricky | 任性 | headstrong | 坚定 | firm |
| 善良 | kind | 踏实 | surefooted | 灵活 | flexible | 自私 | selfish |
| 自信 | confident | 急躁 | impatient | 软弱 | weak | 勤劳 | industrious |
| 活泼 | lively | 狭隘 | narrow | 爽快 | straightforward | 开朗 | cheerful |
| 粗暴 | rough | 虚伪 | hypocritical | 卑鄙 | despicable | 功利 | utilitarian |
| 变态 | abnormal | 友好 | friendly | 诚实 | honest | 愚蠢 | stupid |
| 热情 | passionate | 幽默 | humorous | 悲观 | pessimistic | 贪婪 | greedy |
| 世俗 | vulgar | 从容 | calm | 丑恶 | ugly | 无私 | selfless |
| 刻薄 | mean/ harsh | 奢侈 | luxury | 敏捷 | agile | 高尚 | noble |

*Note*: Self *(Does This Describe You?),* Mother *(Does This Describe Your Mother?),* Grandmother *(Does This Describe Your Grandmother?),* Famous author *(Does This Describe Luxun?)* Students could respond Yes or No to each word. Condition order was balanced across students by using a Latin Square design.

Table S3 *Study 3: Word List by Target Condition*

|  | Self |  | Mother |  | Luxun |  | Grandmother |
| --- | --- | --- | --- | --- | --- | --- | --- |
| 豁达 | open-minded | 虚妄 | false | 古板 | old fashioned | 拘谨 | overcautious |
| 无私 | selfless | 悲观 | pessimistic | 阴险 | insidious | 宽厚 | kind |
| 细腻 | exquisite | 庸俗 | philistine | 下流 | downstream | 孝顺 | filial piety |
| 诚恳 | cordiality | 狡猾 | cunning | 粗暴 | rough | 庄重 | solemn |
| 好学 | studious | 野蛮 | brutal | 卑鄙 | mean | 无畏 | fearless |
| 忠诚 | loyal | 顽固 | stubborn | 软弱 | weak | 慈祥 | amiable |
| 理智 | reason | 多才 | versatile | 消极 | negative | 忠厚 | guileless |
| 灵敏 | sensitive | 愉快 | happy | 克制 | restraint | 勤奋 | diligent |
| 温和 | mild | 要强 | be strong | 敬业 | dedication | 果敢 | dare |
| 勇敢 | brave | 独创 | originality | 出色 | outstanding | 完美 | perfect |
| 成熟 | mature | 威严 | majesty | 可信 | trustworthy | 朴素 | simple |
| 豪爽 | forthright | 散漫 | undisciplined | 昏庸 | fatuous | 泼辣 | pungent |
| 伟大 | great | 幼稚 | naive | 俭朴 | economical | 慎重 | cautious |
| 自卑 | inferiority | 多情 | sentimental | 卑贱 | lowly | 纯洁 | pure |
| 孤僻 | withdrawn | 乖张 | arrogance | 老土 | lag | 谦虚 | self-effacing |
| 执拗 | headstrong | 恍惚 | absent-minded | 轻狂 | mad | 老实 | honest |
| 油滑 | slippery | 寒酸 | shabby | 清高 | clear high | 爱国 | patriotic |
| 狡诈 | deceitful | 仁厚 | gracious | 强硬 | tough | 友好 | amicable |
| 怯懦 | cowardice | 仗义 | righteousness | 聪慧 | intelligent | 粗俗 | vulgar |
| 不孝 | unfilial | 率直 | straightforward | 友善 | friendly | 偏激 | extreme |
| 做作 | affectation | 端庄 | dignified | 节俭 | frugal | 世故 | sophisticated |
| 闲散 | idle | 文静 | quiet | 坦荡 | magnanimous | 高傲 | supercilious |
| 谦卑 | humble | 胆小 | timid | 凶残 | merciless | 窝囊 | burst |
| 懒惰 | lazy | 机警 | alert | 谦让 | polite | 粗心 | thoughtless |
| 功利 | utility | 纯真 | innocent | 憨厚 | thick | 懦弱 | cowardly |
| 古怪 | strange | 爽快 | readily | 热诚 | passionate | 笨拙 | clumsy |
| 蛮横 | outrageous | 开朗 | cheerful | 朴实 | plain | 浮躁 | impetuous |
| 颓废 | decadent | 高尚 | noble | 虔诚 | devotion | 肤浅 | superficial |
| 奢侈 | extravagant | 灵巧 | dexterity | 机灵 | smart | 狠毒 | vicious |
| 残忍 | brutality | 正直 | upright | 沉着 | calm | 马虎 | careless |
| 猖狂 | rampant | 慷慨 | generous | 开明 | enlightened | 滑稽 | funny |
| 狭隘 | narrow | 聪明 | clever | 自立 | self-reliance | 虚荣 | vanity |
| 无情 | ruthless | 正经 | serious | 勤劳 | industrious | 武断 | arbitrary |
| 恬静 | tranquility | 独断 | dictatorial | 圆滑 | sleek | 无趣 | insipid |
| 骄傲 | proud | 虚心 | modest | 细心 | careful | 狂妄 | frantic |
| 柔顺 | compliance | 可爱 | lovely | 爽朗 | hearty | 险恶 | sinister |
| 踏实 | steadfast | 亲切 | warm | 公正 | just | 凶恶 | fiendish |
| 自爱 | self love | 可靠 | reliable | 坚强 | strong | 恶毒 | malevolence |
| 耐心 | patience | 积极 | positive | 自觉 | conscious | 无耻 | shameless |
| 仔细 | discreet | 迟钝 | slow | 独立 | independent | 残酷 | cruel |
| 木讷 | dull | 怪异 | weird | 孤傲 | alone | 大方 | liberal |
| 草率 | sloppy | 没趣 | uninteresting | 莽撞 | collision | 好客 | hospitable |
| 荒唐 | absurd | 轻佻 | frivolous | 势利 | snobbish | 健谈 | talkative |
| 浅薄 | shallow | 平庸 | mediocre | 羞怯 | shy | 博学 | learned |
| 直率 | candour | 消沉 | depressed | 呆板 | rigid | 善变 | fickle |
| 脆弱 | fragile | 轻浮 | skittis | 骄横 | arrogant | 潇洒 | chic |
| 仁慈 | benevolent | 自满 | complacent | 倔强 | unbending | 厚道 | sincere |
| 稳重 | steady | 小气 | parsimonious | 吝啬 | stingy | 清廉 | incorruptible |
| 仁义 | benevolence | 凶狠 | fierce | 伪善 | hypocritical | 温顺 | docile |
| 风趣 | humor | 冷酷 | cold | 专横 | imperious | 贤良 | virtue |

*Note.* This word list used in Yang and Huang (2007). Self *(Does This Describe You?),* Mother *(Does This Describe Your Mother?),* Grandmother *(Does This Describe Your Grandmother?),* Famous author *(Does This Describe Luxun?)* Students could respond Yes or No to each word.

**Studies 1 to 3: Descriptive analyses**

**(IOS, Incidental Recall, and ERP Measures are Distinct)**

We examined the association among our measures using correlational analyses. As detailed next, suggest that IOS scores, incidental recall, and ERP responses were not consistently related. We interpret these results to mean that our three measures captured different aspects of inclusion of others in the self. Grandmother IOS score and grandmother-related recall were weakly positively related in two studies (Study 1 *r*(66) = .29, *p* = .02; Study 3 *r*(117) = .28, *p* = .02) and weakly negatively related in one study (Study 2 *r*(117) = -.18, *p* = .047). Mother IOS scores and mother-related recall were unrelated (*p*s > .50). In Study 3, mother IOS scores, mother-related remember responses, and P3 amplitude of mother-related encoding were unrelated, *p*s > .50, , while P3 amplitude of grandmother-related encoding was not related to grandmother IOS scores or remember responses, *ps* > .25.

**Study 2 Supplemental Analyses**

**Gender.** We found no gender difference in the age (*t*(54) = .94, *p* = .35) or number of years that LB children were left behind (*t*(54) = -.45, *p* = .66). We found a target X gender interaction on the IOS (*F*(1, 113) = 8.53, *p* =.004), girls had higher mother-included-in-self scores than boys (*t*(115) = -2.40, *p* = .02). We did not find a gender main or interaction effect (with target or LB status, *p*s > .50). Hence, we only included gender as a covariate in our IOS analyses.

**Site effects.** We did not control for site in our IOS analyses as we found no site differences in the age that LB children were left behind (*t*(54) = .17, *p* = .86), the number of years they were LB (*t*(54) = .08, *p* = .94), their IOS (*F*(1, 114) = 3.47, *p*= .065), their IOS by target, *F*(1, 114) = 0.78, *p* = .38 or their IOS by LB-status *F*(1, 113) = .08, *p* = .78). We controlled for site in our incidental recall analyses as we found a site main effect on recall (*F* (1, 113) = 13.77, *p* <.001, η^2^_p_ *=* .11), which was moderated by a site by target interaction (*F*(3, 111) = 5.96, *p* = .001, η^2^_p_ *=*.14). Sites did not differ in control target recall (*t*(116) = -1.58, *p* = .12) but Southwest University students were better at self (*t*(116) = 2.55, *p* = .01), mother (*t*(116) = 12.44, *p* < .001), and grandmother (*t*(116) = 2.58, *p* = .011) recall.

**Detailed analyses of incidental recall.** Students had better recall of adjectives linked to self (*t*(116) = 5.32, *p* < .001) and mother (*t*(116) = 3.06, *p* = .003) than adjectives linked to author control. They were as likely to recall adjectives linked to grandmother and to author control (*t*(116) = -.45, *p* = .65).

**Study 3 Supplemental Analyses**

Table S4

*Study 3: Mean (SD) Latency (Reaction Time in Milliseconds) to Remember (R) Response for Words Incidentally Linked to Self, Mother, Grandmother, and a Celebrity among Formerly Left Behind (LB) and Never Left Behind (NLB) Students*

| LB Status | Reaction Time to *R* response | | | | Row Effect Size η^2^_p_ |
| --- | --- | --- | --- | --- | --- |
|  | Self | Mother | Grandmother | Celebrity |  |
| Formerly LB | 1476.22 (306.84) | 1531.19 (317.14) | 1511.25 (312.20) | 1553.96 (309.85) | .076 |
| Never | 1705.57 (370.35) | 1764.67 (349.55) | 1756.33 (366.39) | 1824.92 (395.67) | .165 |
| Column Effect Size η^2^_p_ | .106 | .113 | .119 | .131 |  |

**Exploratory Remember (R) and Know (K) Reaction Time Analyses**. Remember judgments showed a main effect of LB status (*F*(1, 43) = 6.46, *p*= .015, η^2^_p_ = .131) and target (*F*(3, 41) = 3.57, *p* = .022, η^2^_p_ = .207) and no status by target interaction (*F*(3, 41) = 0.22, *p* = .882). Formerly LB students (*M* = 1518.16, *SE* = 68.85) were faster to respond remember than NLB (*M* = 1762.87, *SE* = 67.34). Students were as fast in judging words incidentally linked to self (*M* = 1590.90, *SE* = 50.82), mother (*M* = 1647.93, *SE* = 49.82), and grandmother (*M* = 1633.79, *SE* = 50.85) and slower in judging words incidentally linked to celebrity (*M* = 1689.44, *SE* = 53.14) rather than self, *p* = .010. Know judgments showed no significant main effect or interaction effects, *p*s > .08.

**K response Analysis**. Like van den Bos, and colleagues (2010) we found that target mattered (*F*(3, 41) = 6.16, *p =* .001, η^2^_p_ *=* .311). Students were less likely to report knowing they had seen adjectives related to the self than adjectives related to mother (*F*(1,43) = 10.89, *p* = .002, η^2^_p_ *=* .202) grandmother (*F*(1, 43) = 5.22, *p* = .027, η^2^_p_ *=* .108) or a celebrity (*F*(1, 43) = 14.58, *p* < .001, η^2^_p_ *=* .253). LB status did not matter (*F*(1, 43) = 2.31, *p =* .136). *Figure S1. Study 3 Know (K) responses for words incidentally linked to a target by left-behind status.* Error bars represent 95% confidence intervals.

**Self-Referent Target Effect at P1, N1, P2, N2, and LPP**

ERP captures novel or mismatch detection at P1, N1, and P2 (Evans & Federmeier, 2007; Luck, Woodman, & Vogel, 2000), early attention allocation and stimulus evaluation at P2 (Hillyard & Anllo-Vento, 1998) and emotion processing at LPP (Fields & Kuperberg, 2012; Liu, et al, 2012; Yen, Chen, & Liu, 2010). We had no clear LB-related prediction for these components but self-related processing has sometimes been linked to decreased P1, N1 and N2, enhanced P2 (Fan et al., 2016; Fields & Kuperberg, 2012) and LPP (Fields & Kuperberg, 2012). We explored these regions, looking at the scalp regions in which each has maximum amplitude (following Auerbach et al., 2016; Tacikowski & Nowicka, 2010). We analyzed P1 (100-150ms) using bilateral occipital electrodes (Oz, O1, and O2, Figure S2 Panel A shows mean amplitudes), N1 (140-190ms) and N2 (270-320ms) using bilateral parietal-occipital sites (PO7 and PO8, Figure S2 Panel B shows mean amplitudes). We analyzed P2 (200-300ms) using frontal-central electrodes (FCz, FC3, FC4, Figure S2 Panel C) and LPP (600-1000ms) using fronto-central sites (Fz, FCz, Cz, Figure S2 Panel D).

**P1.** We found no significant main effect of target (*F*(3, 41) = .82, *p* = .489, η_p_^2^=.057) and no significant LB status effect (LB main *F*(1, 43) = 0.01, *p* = .921, η_p_^2^ < .001, LB x target interaction *F*(3, 41) = 1.64, *p* = .194, η_p_^2^ = .107).

**N1.** We found no significant main effect of target (*F*(3, 41) = 1.73, *p* = .176, η_p_^2^ = .112) and no significant LB status effect (LB main *F*(1, 43)= 1.03, *p* = .316, η_p_^2^ = .023, LB x target interaction *F*(3, 41) = 1.02, *p* = .393, η_p_^2^ = .068).

**P2.** We found a significant LB status by target interaction (*F*(3, 41) = 8.88, *p* < .001, η_p_^2^ = .394) that moderated the main effect of target (*F*(3, 41) = 0.62, *p* = .605, η_p_^2^ = .043) and LB status (*F*(1, 43) = 0.14, *p* = .709, η_p_^2^= .003). To decompose this interaction, we looked separately at the effect of target on NLB and LB students. Target mattered for NLB students (*F*(3, 41) = 6.95, *p* = .001, η*_p_*^2^ = .337), not LB students *F*(3, 41) = 2.64，*p* = .062, η_p_^2^ = .162. Specifically, NLB students had the same amplitude response to self and mother (*p* = .372) and to self and grandmother (*p* = .101), and higher amplitude response to mother than to grandmother (*p* = .001) or celebrity (*p* < .001), and higher amplitude response to self than to celebrity (*p* = .038). LB students had the same amplitude response to self and mother (*p* = .994) and to self and grandmother (*p* = .134). They had larger amplitude response to grandmother than mother (*p* = .020). But we cannot be sure of stability of these results given that their self and celebrity P2 responses did not differ (*p* = .099).

**N2.** We found no significant effect of target (main *F*(3, 41) = .30, *p* = .827, η_p_^2^ = .021) or LB status (LB main *F*(1, 43)= 0.05, *p* = .825, η_p_^2^ = .001, LB x target interaction *F*(3, 41) = 1.67, *p* = .189, η_p_^2^ = .109).

**LPP**. Target mattered (main effect *F*(3, 41) = 3.03, *p* = .040, η_p_^2^=.182) but LB status did not (main *F*(1, 43)= 0.03, *p* = .872, η_p_^2^ = .001, LB x target interaction *F*(3, 41) = 1.92, *p* = .141, η_p_^2^ = .123). Regarding target, Self-Condition evoked larger amplitude than Celebrity-Condition, *p* = .030.


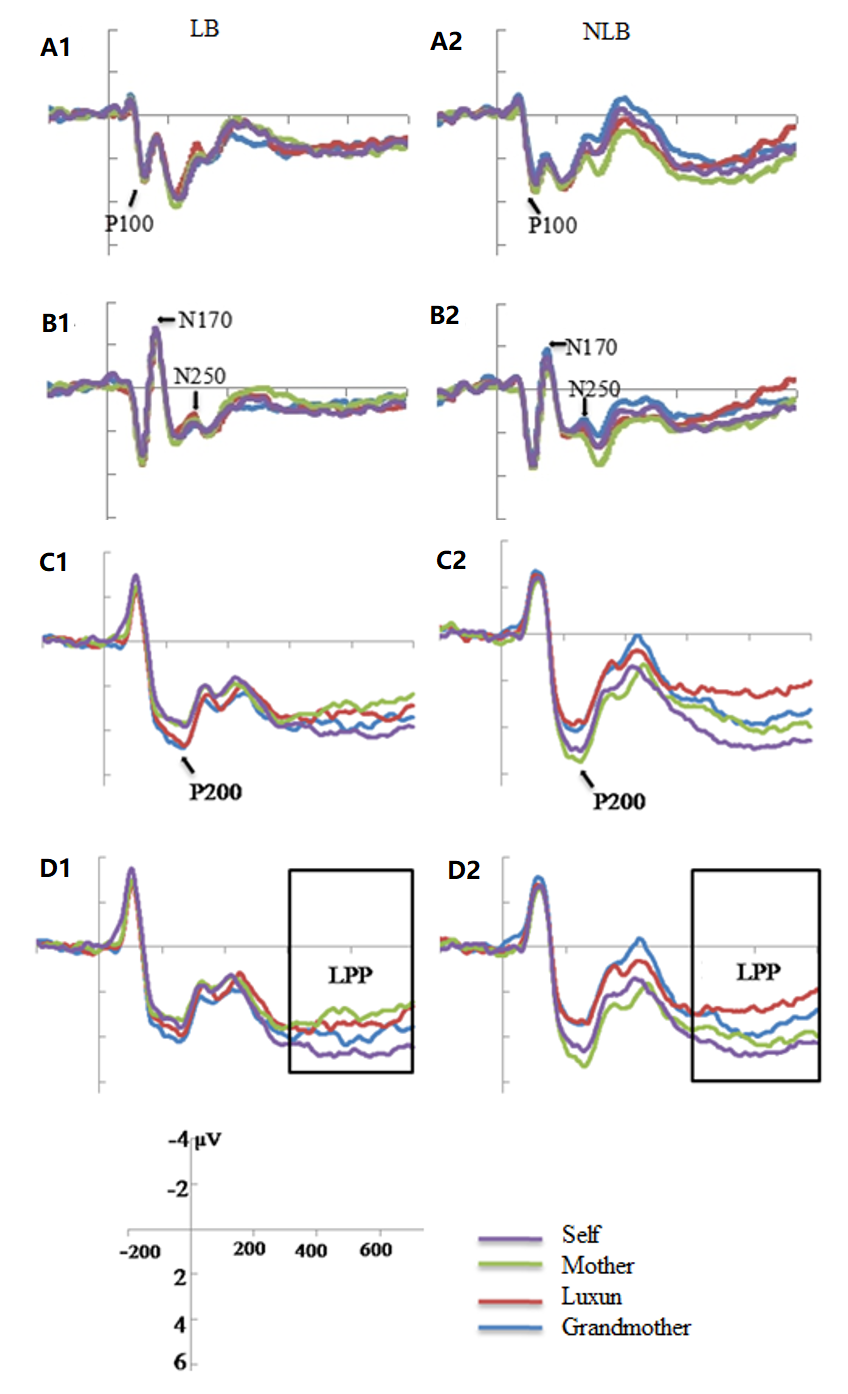


Figure S2. Illustration of grand averaged waveforms (P1, N1, P2, N2, LPP) across conditions for formerly left-behind (LB) and never left-behind (NLB) undergraduates

**Supplemental Materials** **References**

Auerbach, R., Bondy, E., Stanton, C., Webb, C., Shankman, S., & Pizzagalli, D. (2016). Self-referential processing in adolescents: Stability of behavioral and ERP markers. *Psychophysiology*, *53*(9), 1398-1406. doi: 10.1111/psyp.12686

Evans, K. & Federmeier, K. (2007). The memory that's right and the memory that's left: Event-related potentials reveal hemispheric asymmetries in the encoding and retention of verbal information. *Neuropsychologia, 45*(8), 1777-1790. doi: 10.1016/j.neuropsychologia.2006.12.014

Fan, W., Zhong, Y., Li, J., Yang, Z., Zhan, Y., Cai, R., & Fu, X. (2016). Negative emotion weakens the degree of self-reference effect: Evidence from ERPs. *Frontiers in Psychology*, *7*, 1408. doi: 10.3389/fpsyg.2016.01408

Fields, E. & Kuperberg, G. (2012). It's all about you: An ERP study of emotion and self-relevance in discourse. *Neuroimage*, *62*(1), 562-574. doi: 10.1016/j.neuroimage.2012.05.003

Hillyard, S., & Anllo-Vento, L. (1998). Event-related brain potentials in the study of visual selective attention. *Proceedings of the National Academy of Sciences*, *95*(3), 781-787. doi: 10.1073/pnas.95.3.781

Liu, Y., Huang, H., McGinnis-Deweese, M., Keil, A., & Ding, M. (2012). Neural substrate of the late positive potential in emotional processing. *The Journal of Neuroscience, 32*(42), 14563-14572. doi: 10.1523/JNEUROSCI.3109-12.2012.

Luck, S., Woodman, G., & Vogel, E. (2000). Event-related potential studies of attention. *Trends in Cognitive Sciences*, *4*(11), 432-440. doi:10.1016/S1364-6613(00)01545-X

Tacikowski, P., & Nowicka, A. (2010). Allocation of attention to self-name and self-face: An ERP study. *Biological Psychology, 84*(2), 318-324. doi: 10.1016/j.biopsycho.2010.03.009.

van den Bos, M., Cunningham, S. J., Conway, M. A., & Turk, D. J. (2010). Mine to remember: The impact of ownership on recollective experience. *The Quarterly Journal of Experimental Psychology*, *63*(6), 1065-1071.

Yen, N., Chen, K., & Liu, E. (2010). Emotional modulation of the late positive potential (LPP) generalizes to Chinese individuals. *International Journal of Psychophysiology, 75*(3), 319-325. doi: 10.1016/j.ijpsycho.2009.12.014.
